# Testotoxicosis: Report of Two Cases, One with a Novel Mutation in LHCGR Gene

**DOI:** 10.4274/jcrpe.2067

**Published:** 2015-08-31

**Authors:** Bahar Özcabı, Feride Tahmiscioğlu Bucak, Serdar Ceylaner, Rahşan Özcan, Cenk Büyükünal, Oya Ercan, Beyhan Tüysüz, Olcay Evliyaoğlu

**Affiliations:** 1 İstanbul University Cerrahpaşa Faculty of Medicine, Department of Pediatric Endocrinology, İstanbul, Turkey; 2 Intergen Genetics Centre, Ankara, Turkey; 3 İstanbul University Cerrahpaşa Faculty of Medicine, Department of Pediatric Surgery, İstanbul, Turkey; 4 İstanbul University Cerrahpaşa Faculty of Medicine, Department of Pediatric Genetic, İstanbul, Turkey

**Keywords:** Testotoxicosis, microlithiasis, anastrozole, bicalutamide, cyproterone acetate, ketoconazole

## Abstract

Testotoxicosis is a rare disorder which presents as isosexual peripheral precocious puberty in males. Despite the pattern of autosomal dominant inheritance, sporadic cases also may occur. Due to activating mutation in luteinizing hormone (LH))/choriogonadotropin receptor (LHCGR) gene, early virilization and advancement in bone age are common with increased serum testosterone levels above adult ranges, despite low LH and follicular-stimulating hormone (FSH) levels. There are different treatment regimens, such as combination of bicalutamide (antiandrogen agent) and a third-generation aromatase inhibitor, that are reported to be well-tolerated and successful in slowing bone age advancement and preventing progression of virilization. We report here two patients who presented with peripheral precocious puberty and an activating mutation in the LHCGR gene: one with a family history and previously determined mutation and the other without family history and with a novel mutation (c.830G>T). Combination of bicalutamide+anastrozole was ineffective in slowing pubertal progression and bone age. Short-term results were better with ketoconazole.

## INTRODUCTION

First described by Schedewei et al (1), familial male-limited precocious puberty is a rare, autosomal dominant form of gonadotropin-independent precocious puberty in males. Sporadic cases were also reported (2,3). In this condition, due to activating mutations in luteinizing hormone (LH)/choriogonadotropin receptor gene (LHCGR), testicular steroidogenesis and spermatogenesis are stimulated (1,2,4).

This disorder was also called “testotoxicosis” by Rosenthal et al (2) The age of onset is usually 2-4 years. Unilateral or bilateral testicular growth is observed. Nodular Leydig cell hyperplasia can be observed and seminiferous tubular development is usually sufficient to allow spermatogenesis (2,3,5,6). Due to activating mutation in the LHCGR gene, an extremely muscular body build, accelerated growth and advanced bone age are commonly observed. Testosterone levels are in or above the adult male range with low LH and follicular-stimulating hormone (FSH) levels. Central precocious puberty typically follows over time. After the maturation of hypothalamo-pituitary-testicular axis, normal adult function appears to range from normal paternity to reduced testicular volume or oligospermia (7).

Although the reported mutations in familial and sporadic cases had a genetic heterogeneity, they were usually sited in exon 11 (3,4,8,9,10,11). However, further studies revealed that there were a considerable number of cases, especially sporadic forms, whose mutations were sited in exons other than 11 (10,12). In order to reduce the synthesis and action of androgens, several agents such as medroxiprogesterone, ketoconazole, cyproterone have been used for treatment. Aromatase inhibitors are also included in treatment regimens for slowing of bone age advancement. In recent studies, different combinations of new generation aromatase inhibitors and anti-androgen agents such as bicalutamide have been reported and recommended for the treatment of difficult cases (13,14,15,16, 17,18,19,20,21,22,23).

We report here two patients who presented with an activating mutation in the LHCGR gene: one familial with a previously reported mutation and one sporadic with a novel mutation (c.830G>T), in whom combination of bicalutamide+anastrozole treatment was ineffective.

## CASE REPORTS

### Patient 1

**Figure 1:**
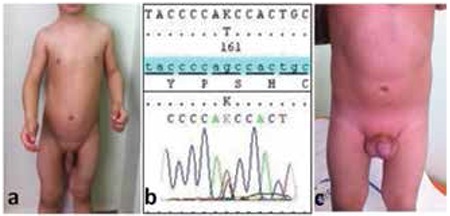
Note the enlarged external genitalia, extremely muscular body build (a) and novel mutation c.830G>T (p.S277I) in the LHCGR gene (b) of patient 1 and also the enlarged external genitalia of patient 2 (c)

A 1.4-year-old boy was admitted with complaints of pubic hair development, penile enlargement, linear growth acceleration, acne and increased aggressive behavior. His mother had become aware of enlargement of the genitals by 6 months of age. He had healthy parents non-consanguineously married and there was no family history of precocious puberty. At physical examination, his height was 96 cm (+5.38 standart deviation score (SDS)) and weight was 17 kg (+2.0 SDS). He had an extremely muscular body build, a deepened voice and acne. Penile stretch length was 13 cm (>2 SDS); the left testicular volume was 3 mL and the right was 4 mL. Pubic hair was appropriate for Tanner stage 3 (Figure 1a). He did not have any café au lait spots.

Bone age was 4 years by the Greulich-Pyle method. Mid-parental height (MPH) SDS was 0.83. Clinical, auxological and endocrinological findings at admission are summarized in Table 1. The patient’s serum testosterone level was very high and gonadotropin-releasing hormone (GnRH) stimulation test revealed a prepubertal response. Thyroid function tests (free triiodothyronine (T3): 4.4 pg/dL; free thyroxine (T4): 1.1 ng/dL; thyroid-stimulating hormone (TSH): 2.5 mIU/L) and adrenal cortex hormone levels (dehydroepiandrosterone sulfate (DHEA-SO4): 37.2 µg/dL; 17-α-hydroxyprogesterone (17-OHP): 0.9 ng/dL; androstenedione: 0.5 ng/mL; cortisol: 10.7 µg/dL) were within normal ranges. Peripheral precocious puberty findings with a testicular volume of 4 mL suggested a tumor secreting beta-human chorionic gonadotropin (hCG) or a testicular tumor secreting testosterone; an activating mutation in the LH receptor and McCune Albright syndrome were also considered. Abdominal magnetic resonance imaging (MRI) revealed normal findings. A low serum beta-hCG (<1.20 mIU/L) level excluded presence of a beta-hCG-secreting tumor. He did not have any abnormality in bone scintigraphy screening. A testicular ultrasonography showed a solid mass of a size of 2x2 mm in the right testicle. A surgical intervention was performed. During the operation, the testis was mobilized by inguinal incision and a 2x2 mass was detected. In cold ischemia conditions, the mass was excised by testis-saving procedure and sent for histopathological evaluation. Pathological examination revealed nodular Leydig cell hyperplasia. After surgery, serum total testosterone level did not decline. Anti-androgen treatment with bicalutamide (50 mg/day) and aromatase inhibition with anastrozole (1 mg/day) were initiated. A normal testosterone level could not be obtained and another surgery was planned. The same inguinal incision was performed and vascular pedicle was secured in cold ischemia conditions. The mass was not localized by visual examination. Intraoperative ultrasonography demonstrated a 3x3 mm mass and with the guidance of the probe, the mass was found and excised by another testis-saving procedure. Histopathological examination revealed the diagnosis of nodular Leydig cell hyperlasia again. Since the patient met the criteria for the diagnosis of testotoxicosis, a genetic analysis for LHCGR gene was performed. Sequence analysis of all coding regions and exon-intron boundaries were done by in house designed primers using Sanger sequencing technique and a novel mutation c.830G>T (p.S277I) (heterozygous) was described (Figure 1b). In silico evaluation tools including Mutation taster, SIFT and Polyphen 2 predict this variant as a disease-causing mutation. Parental analysis was normal, then this variation was most probably a de novo pathogenic variant. Screening of 200 healthy people for this variant was done and no one had this mutation.

Cyproterone acetate was added to the treatment regimen because the suppression of pubertal progression and serum testosterone levels was not sufficient, but it was withdrawn in one month because of increases in liver enzymes which could not be associated with another cause; liver enzymes became normal after the cessation of the drug.

In the 20th month of treatment, GnRH stimulation revealed a pubertal response and a GnRH analogue was added to treatment. In his follow-up at age 3.8 years, despite bicalutamide (100 mg/day), anastrozole (2 mg/day) and GnRH analogue treatments, sufficient suppression of puberty and decline in serum testosterone levels were not achieved (Table 2). Antiandrogen treatment with bicalutamide (androgen receptor antagonist) was changed to ketoconazole (androgen synthesis inhibitor) in a dose of 10 mg/kg/day. A treatment regimen of ketoconazole, anastrozole and a GnRH analogue led to a decline in serum testosterone level from 900 ng/dL to 490 ng/dL on the 3rd day of the treatment. At the 3rd month of ketoconazole (15 mg/kg/d) treatment, serum testosterone level decreased to 125 ng/dL without any sign of side effects; whether this benefit will be sustained or not needs to be evaluated in the long term.

### Patient 2

Patient 2 was admitted at the age of 1.5 years with complaints of pubic hair development, penile enlargement and linear growth acceleration. His mother had become aware of the symptoms 1 month before admission. He had healthy parents with a non-consanguineous marriage, but in the family history, there were other cases of precocious puberty.

At physical examination, the patient’s height was 94.2 cm (+3.1 SDS) and his weight was 16.5 kg (+2.9 SDS). Stretched penile length was 6 cm (>2 SDS); left testicular volume was 3 mL and right testicular volume was 4 mL. Pubic hair was Tanner stage 2 (Figure 1c). He did not have any café au lait spots.

His bone age was 3.5 years by the Greulich-Pyle method. MPH SDS was 1.3. Clinical, auxological and endocrinological findings at admission are summarized in Table 1. Similar to the first patient, serum testosterone level was very high and GnRH stimulation test revealed a prepubertal response. Thyroid function tests were within normal ranges (free T3: 5.2 pg/dL, free T4: 1.1 ng/dL, TSH: 3.2 mIU/L). Baseline 17-OHP level was 6.1 ng/dL and peak level was 11.6 ng/mL by an adrenocorticotropic hormone stimulation test. CYP21 gene analysis revealed no mutation. He had normal abdominal MRI. Low serum beta-hCG (<1.2 mIU/L) level excluded an hCG-secreting tumor. There was no tumoral mass in testicular ultrasound, but it revealed bilateral hydrocele and microlithiasis. Bone scintigraphy was normal. A combination of bicalutamide (50 mg/day)+anastrozole (1 mg/day) treatment was started. At the genetic analysis for testotoxicosis, sequence analysis of all coding regions and exon-intron boundaries of LHCGR gene was done by in house designed primers using Illumina Miseq Next Generation sequencing technique and a known mutation c.1118C>T (p.A373V) (heterozygous) was determined. Analyses in the parents showed that the mother also had this mutation.

Despite the treatment, as also was the case in the former patient, sufficient suppression of puberty characteristics and decline in serum testosterone levels were not achieved at 24 months of treatment (Table 2). Bicalutamide treatment was switched to ketoconazole (10 mg/kg/day) and anastrozole (2 mg/day) treatment was continued. Serum testosterone level declined to 10 ng/dL from 490 ng/dL on the third day of treatment. At the end of the first month of this combination treatment, as total testosterone level increased to 23 ng/dL, the ketoconazole dose was raised to 15 mg/kg/day. Gonadotropin levels were still in prepubertal ranges and total testosterone levels did never exceeded 23 ng/dL after the dose increment. At the 6th month of follow-up with ketoconazole and anastrozole, serum testosterone level was 15 ng/dL without any side effect and bone age was still appropriate for age 5.5 years.

## DISCUSSION

Testotoxicosis becomes usually symptomatic by 2-4 years of age; however, in some cases, the clinical signs and laboratory findings of puberty appear earlier (2,3). Teles et al (9) reported a healthy 10-month-old boy investigated because of an older brother with familial testotoxicosis. The same heterozygous mutation Thr577Ile was determined and the bone age was found to be advanced. In this patient, despite lack of virilization signs, cyproterone acetate was started at the age of 1.3 years in order to decrease testosterone levels and growth velocity. In our first patient, pubic hair development and penile enlargement had started at the age of six months and, to our knowledge, he is the youngest patient with virilization symptoms. We can speculate that this early and severe clinical aspect was related to the novel c.830G>T (p.S277I) mutation in the LHCGR gene.

In patients with testotoxicosis, in addition to clinical and laboratory investigations, genetic analyses are beneficial for diagnosis and follow-up. Several mutations were reported in Caucasian, Afro-American and Brazilian populations. There has been genetic heterogeneity, but mutations were usually sited in exon 11 (3,4,8,9,10,11). However, further studies revealed that there were a considerable number of cases, especially sporadic forms, in which mutations were commonly sited in exons other than 11 (10,12). In patient 1, a novel mutation c.830G>T (p.S277I) (heterozygous) was determined in exon 9 of LHCGR gene and he is a sporadic case. According to his family history, patient 2 probably had familial testotoxicosis, but unfortunately, the other family members with probable testotoxicosis did not give permission for investigation. Our second patient and his mother had the mutation c.1118C>T (p.A373V) (heterozygous) previously reported by Gromoll et al (11) in 11 exon of LHCGR gene. Some studies have attempted to establish an association between phenotype and genotype (3,10,11). Signs of precocious puberty appeared at age 17 months in our patient 2, while the patient who had the same mutation and was reported by Gromoll et al (11) had signs at the age of 5 years. Although presentation ages were different, ketoconazole treatment was effective in both cases in the short term.

Independently from treatment, testotoxicosis can cause testicular changes. In biopsy specimens from patients with testotoxicosis, Gondos et al (5) observed that the morphologic changes indicated premature differentiation of all of the major testicular cell types. Leydig cells demonstrated nuclear and cytoplasmic features characteristic of fully differentiated steroidogenic cells; germ cells at all stages of spermatogenesis were present, but their maturation was disorganized and spermatids had structural abnormalities. In 1998, Martin et al (24) reported testicular seminoma in an adult patient. He was diagnosed at the age of 27 months and had the A578G mutation in LHCGR gene. However, seminoma is not the only cause of solid mass in testes of affected patients. Leschek et al (6) reported nodular Leydig cell hyperplasia in a patient with testotoxicosis who had A564G mutation. Thus, our first patient is the second reported case with nodular cell hyperplasia which might be due to overstimulation of Leydig cells. Testicular microlithiasis determined in patient 2 is an uncommon condition characterized by calcium deposits in the lumina of seminiferous tubules. These intratesticular calcifications appear as bright, 2- to 3-mm echogenic foci on testicular ultrasound. It might be a benign condition with a potential for malignancy. It is reported that this ultrasonographic sign can occur in many conditions such as germ cell tumors, epididymitis, cryptorchidism, retractile testis and hypotrophic testis (25,26,27). While observed in some cases (25,26), hydrocele is not a common cause of testicular microlithiasis (27). Microlithiasis has also not been reported in patients with LH-activating mutations; Gougoudi et al (28) reported that testicular microlithiasis co-existed with Leydig cell hyperplasia in Wistar rats. Thus, whether Leydig cell hyperstimulation in our patient is the cause of microlithiasis or not is not known.

In testotoxicosis, there is no consensus for treatment modality and, as shown in Table 3, different regimens have been proposed. Rosenthal et al (13) used oral medroxyprogesterone in two boys and observed distinct decrease in serum total testosterone level and height velocity. One agent which has been widely used is ketoconazole which inhibits adrenal and testicular androgen biosynthesis. Holland et al (14) reported that ketoconazole (3x200 mg/dose) led to a reduced testosterone levels in three affected patients, but after 1-3 months of treatment, a marked pubertal response to GnRH stimulation test was found to occur. This phenomenon could question the use of ketoconazole alone (14). Bertelloni et al (7) treated two patients with cyproterone acetate and 2 patients with ketoconazole. Cyproterone acetate was ineffective in clinical progression. Although ketoconazole reduced testosterone secretion and improved the final height in one patient, the second patient had a short adult height of -2 SDS which was below his target height. Conversely, Soriano-Guillén et al (15) observed that final heights of five patients treated with ketoconazole (16.2 mg/kg/day) were in concordance with their target heights and were significantly higher than their pretreatment predicted heights. Total testosterone levels with treatment were less than 0.5 ng/mL and none of the patients had early activation of the pituitary-gonadal axis. Ketoconazole was well-tolerated; only one patient had a transient and modest increase in serum transaminases (15). The main side effect of ketoconazole is hepatic injury that may lead to cirrhosis (29,30). Other side effects such as interstitial pneumonitis have been also reported (16). Cyproterone acetate which antagonizes androgen action at the receptor level is another agent administered in testotoxicosis (7,17,18). Almeida et al (17) reported a multicentric, retrospective, long-term treatment study in which 10 patients were evaluated. Cyproterone acetate was administered to five patients and ketoconazole to five others. Age of onset of disease and duration of treatment were similar. The authors concluded that long-term treatment with cyproterone acetate or ketoconazole resulted in similar outcomes without serious side effect. However, both agents had limited effectiveness on height.

Further studies revealed that new combinations of aromatase inhibitors+anti-androgen agents such as anastrozole+cyproterone acetate (19) and anastrozole+bicalutamide (20,21,22,23) were effective in reducing virilization and in decreasing testosterone synthesis without important complications. Lenz et al (21) reported two boys treated with anastrozole+bicalutamide. This combination was well-tolerated, effective in preventing progression of virilization and in slowing bone-age advancement with no decrease in linear growth (21). The weakness of all these studies was the small number of patients or short-term outcomes.

Testotoxicosis may be difficult to treat in some cases. Although bicalutamide treatment has been reported as well-tolerated and effective, in the presented 2 patients, it was not successful in reducing serum testosterone levels, bone age advancement and pubertal progression. Short-term laboratory and clinical results of ketoconazole treatment seemed to be more efficacious in these two patients. Long-term studies with large numbers of patients are necessary for evaluation of these agents regarding prognosis, treatment efficacy and long-term effects on adult height, fertility and metabolic parameters.

## Figures and Tables

**Table 1:**
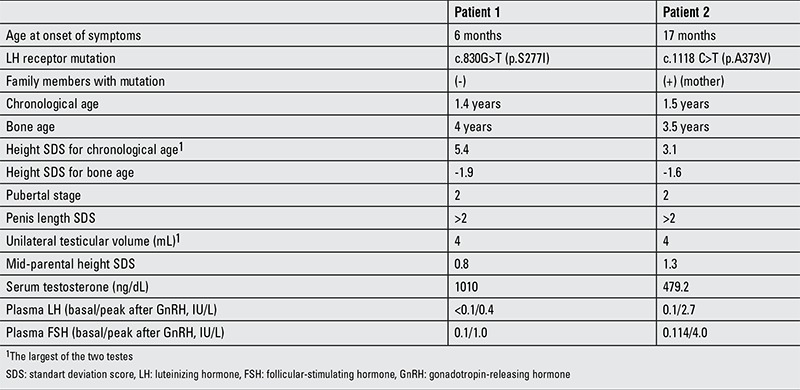
Clinical, auxological and endocrinological data of the patients at admission

**Table 2:**
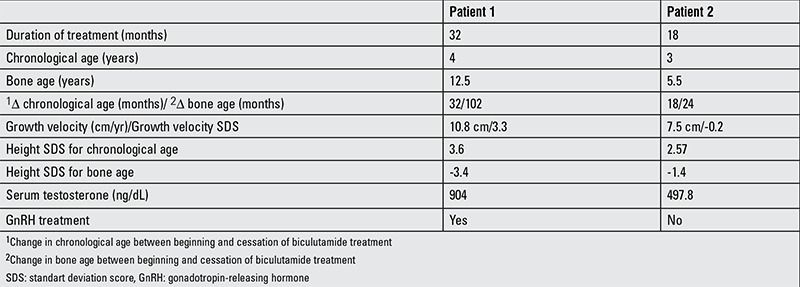
Data at cessation of bicalutamide+anastrozole treatment

**Table 3:**
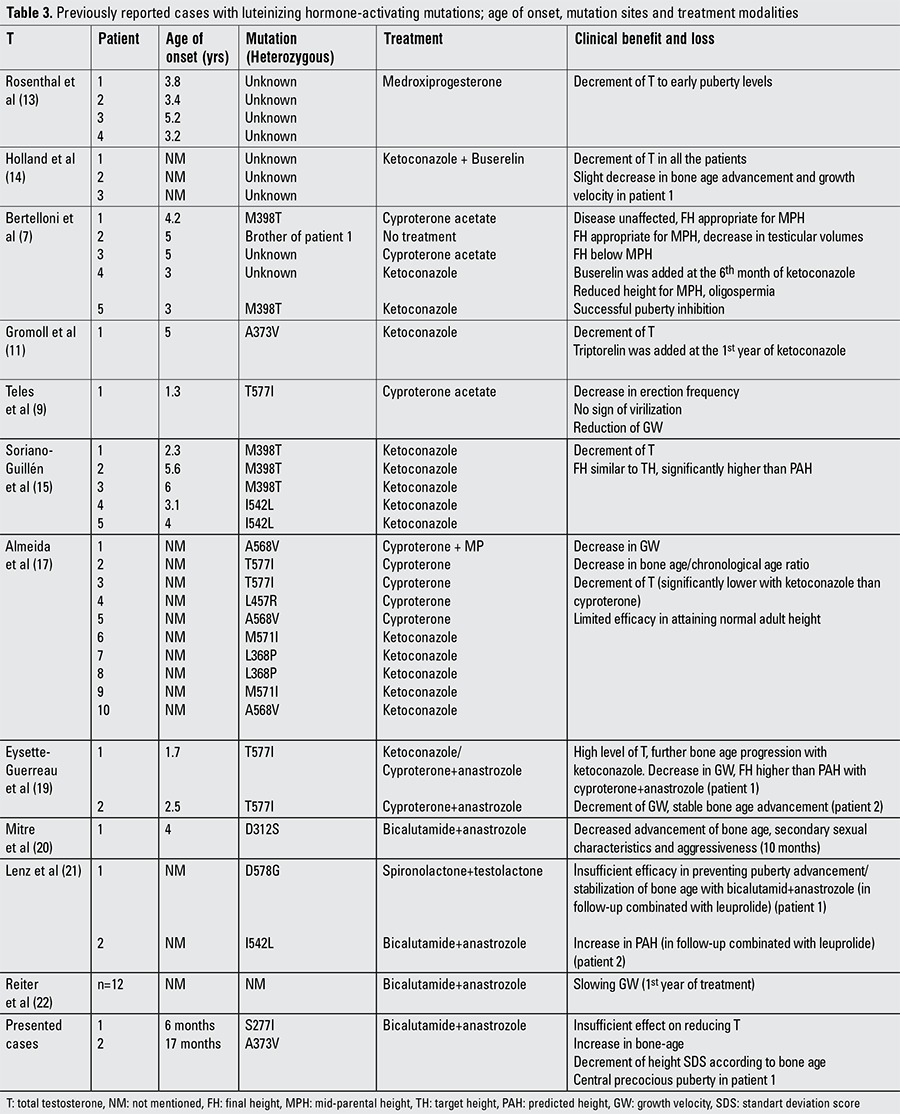
Previously reported cases with luteinizing hormone-activating mutations; age of onset, mutation sites and treatment modalities
